# Interdependent effects of male and female body size plasticity on mating behaviour of predatory mites

**DOI:** 10.1016/j.anbehav.2014.11.017

**Published:** 2015-02

**Authors:** Andreas Walzer, Peter Schausberger

**Affiliations:** Group of Arthropod Ecology and Behavior, Division of Plant Protection, Department of Crop Sciences, University of Natural Resources and Life Sciences, Vienna, Austria

**Keywords:** exploitation competition, interference competition, phytoseiid mites, sexual conflict, polyandry

## Abstract

The adaptive canalization hypothesis predicts that traits with low phenotypic plasticity are more fitness relevant, because they have been canalized via strong past selection, than traits with high phenotypic plasticity. Based on differing male body size plasticities of the predatory mites *Phytoseiulus persimilis* (low plasticity) and *Neoseiulus californicus* (high plasticity), we accordingly hypothesized that small male body size entails higher costs in female choice and male–male competition in *P. persimilis* than *N. californicus*. Males of both species are highly polygynous but females differ in the level of polyandry (low level in *P. persimilis*; medium level in *N. californicus*). We videotaped the mating interactions in triplets of either *P. persimilis* or *N. californicus*, consisting of a virgin female (small or standard-sized) and a small and a standard-sized male. Mating by both small and standard-sized *P. persimilis* females was biased towards standard-sized males, resulting from the interplay between female preference for standard-sized males and the inferiority of small males in male–male competition. In contrast, mating by *N. californicus* females was equally balanced between small and standard-sized males. Small *N. californicus* males were more aggressive (‘Napoleon complex’) in male–male competition, reducing the likelihood of encounter between the standard-sized male and the female, and thus counterbalancing female preference for standard-sized males. Our results support the hypothesis that male body size is more important to fitness in the low-level polyandrous *P. persimilis* than in the medium-level polyandrous *N. californicus* and provide a key example of the implications of sexually selected body size plasticity on mating behaviour.

## Highlights

• We evaluated the effects of body size on mating behaviour in two mite species.
• Small body size reduced the mating success of *Phytoseiulus persimilis* males.
• Mating success of *Neoseiulus californicus* males was unaffected by body size.
• Lower male body size plasticity in *P. persimilis* is sexually selected.

Male body size is a decisive factor for the outcome of male–male competition and female choice in numerous animals (Andersson, 1994, Blanckenhorn, 2005). Commonly, the competitively inferior males are smaller than their rivals, which are also more often selected by the females as mates (Wong & Candolin, 2005). In some species, however, small males have evolved alternative mating tactics such as sneakers, satellites or patrollers, to circumvent direct male–male competition and increase their mating success (Gross, 1996, Taborsky et al., 2008). Small males may also act hyperaggressively against larger rivals (‘Napoleon complex’) and frequently initiate fights (Jenssen et al., 2005, Moretz, 2003). Hyperaggressive behaviour of small males may be adaptive if the probability of winning male–male competition is higher for the initiating than reacting male (Morrell, Lindström, & Ruxton, 2005).

Large dominant males do not always guarantee the highest net benefit for females willing to mate. For example, the direct costs of interacting with dominant, large males arising from harassing may lead to female preference for small males (Qvarnström & Forsgren, 1998). Harassment costs should vary with female phenotype such as body size. Small females have a higher risk of injury by male harassment and may thus prefer small over large mates (Crespi, 1989). Size-assortative female choice may balance the mating success of small and large males. Indirect costs may arise when large males are comparatively poor food providers of offspring (Forsgren, 1997), resulting in female preference for small males (Hakkarainen et al., 1996).

Regarding sexual selection, male body size should be less important to fitness in species in which the costs of being small are counterbalanced by male or female strategies than in species without such compensating mechanisms. The fitness relevance of male body size should in turn be reflected in its plasticity. Strong past sexual selection on male body size should reduce plasticity, according to the adaptive canalization hypothesis. Robustness of male body size against environmental disturbances, preventing large deviations from the optimum male size, is considered an adaptive consequence of canalization (Schmalhausen, 1949, Stearns and Kawecki, 1994, Stillwell et al., 2010, Waddington, 1942). Strikingly, the predictions of the adaptive canalization hypothesis have primarily been used to explain the evolution of body size plasticity in the context of sexual size dimorphism within species (Stillwell et al., 2010). We argue that species- and sex-specific variation in canalization of body size plasticity may also reflect species-specific strengths in past sexual selection arising from different mating systems (Walzer and Schausberger, 2011, Walzer and Schausberger, 2014).

Our study system consisted of the plant-inhabiting predatory mite species *Phytoseiulus persimilis* and *Neoseiulus californicus*, which constitute a natural predator guild in several regions in the Mediterranean Basin (De Moraes, McMurtry, Denmark & Campos, 2004), sharing the two-spotted spider mite, *Tetranychus urticae*, as prey (Walzer & Schausberger, 2011). In both species, both females and males develop from fertilized eggs but the male genome is eliminated during egg development resulting in haploid males (Sabelis, Nagelkerke, & Breeuwer, 2002). Adult males are about one-third smaller than adult females (e.g. Walzer & Schausberger, 2011). The female-biased tertiary sex ratio ranges from 0.6 to 0.8 under optimal conditions and can shift to equality, when the population density increases or prey availability decreases (Sabelis et al., 2002). Both species are highly aggregated at the leaf scale (Walzer, Moder, & Schausberger, 2009), but patchily distributed among leaves within plants (Zhang & Sanderson, 1993). Males actively search for mates, orienting themselves on pheromones released by the females (Amano and Chant, 1978a, Pappas et al., 2005). Male–male fighting has been observed in *P. persimilis* (Enigl & Schausberger, 2004). It is unknown whether *N. californicus* males also engage in contest mate competition. After the mates encounter each other, the female decides whether or not mating takes place. Female choice may manifest as the female readily accepting the potential mate, delaying the mating or completely avoiding the potential mate by running away (personal observation), with the latter indicating female resistance and/or sexual conflict (Gosden & Svensson, 2009). In both species the average mating duration is 2–4 h (Amano and Chant, 1978b, Enigl and Schausberger, 2004, Gotoh and Tsuchiya, 2008). The mates do not stay together after mating, indicating the absence of male postcopulatory guarding behaviour (Amano & Chant, 1978b). Males of both *P. persimilis* and *N. californicus* are highly polygynous but the female mating system differs between the two species (Schausberger et al., 2014, Walzer and Schausberger, 2014). A single mating is sufficient for *P. persimilis* females to reach the maximum lifetime reproductive success (LRS) but females do remate occasionally (Schausberger et al., 2014). In contrast, *N. californicus* females need multiple matings to reach the maximum LRS (Gotoh and Tsuchiya, 2008, Schausberger et al., 2014). Thus, we defined the female mating systems of *P. persimilis* and *N. californicus* as low-level and medium-level polyandry, respectively (Schausberger et al., 2014, Walzer and Schausberger, 2014). Food shortage during juvenile development induces species- and sex-specific adult body size plasticity. In both species female body size is similarly plastic and more plastic than male body size. However, male body size is more plastic in *N. californicus* than *P. persimilis* (Walzer and Schausberger, 2011, Walzer and Schausberger, 2014). Consistent with the adaptive canalization hypothesis, small deviations from standard male body size reduce male LRS of *P. persimilis* but not *N. californicus* (Walzer & Schausberger, 2014).

Here, we tested the predictions that (1) the fitness relevance of male body size plasticity is also reflected in male–male competition and female choice of *P. persimilis* and *N. californicus*, and (2) the costs of small male body size are more effectively counterbalanced in *N. californicus* than *P. persimilis*. To this end, we conducted mating experiments using triplets consisting of a single small and standard-sized male and a small or standard-sized female and characterized the mating behaviour (mate choice, mating latency, duration and frequency) and the intensity and direction of male–male competition and female choice within the triplets.

## Methods

### Species Origin and Rearing

Specimens of *P. persimilis* and *N. californicus* used to found laboratory-reared populations originated from Sicily (Walzer & Schausberger, 2011). The species were reared on separate arenas consisting of plastic tiles resting on water-saturated foam cubes in plastic boxes half-filled with water (for details see Walzer & Schausberger, 2014). To obtain predator eggs for generating small and standard-sized females and males used in experiments, 10 females each of *N. californicus* and *P. persimilis* were randomly taken from the rearing units and placed on separate spider mite-infested bean leaf arenas for egg production (for details see Walzer & Schausberger, 2014).

### Experimental Cages and Arenas

Cages drilled into rectangular acrylic plates were used for generating virgin females and males with different body sizes. Each cage consisted of a cylindrical cell 15 mm in diameter and 3 mm high closed at the bottom by fine gauze and on the upper side by a microscope slide (Schausberger, 1997). White plastic discs (diameter 14 mm) were used as experimental arenas. The chosen disc size provided sufficient space for free movement of the predators (body length ca. 0.3–0.5 mm), but at the same time increased the likelihood of encounter (Walzer and Schausberger, 2013a, Walzer and Schausberger, 2013b). Additionally, the disc size fitted the requirements of the video-tracking software we used, EthoVision XT 8, to distinquish the individuals from system noise without identification errors (Noldus Information Technology b.v., 2010). Each plastic disc was fixed on a metallic cylinder (height 20 mm, diameter 10 mm), which was centrally placed in a cubic plastic box (side length 25 mm) filled with tap water up to the margin of the disc to confine the mites to the arena. Forty spider mite eggs serving as prey for the predators were placed on each disc using a moistened camel's hair brush.

### Generating Small and Standard-sized Females and Males

To get small and standard-sized females and males of *P. persimilis* and *N. californicus*, respectively, eggs were randomly taken from the egg production arenas, placed singly into acrylic cages and provided with either limited (10 for *P. persimilis*, eight for *N. californicus*) or ample (40 for either predator) spider mite eggs as prey. Limited prey supply differed between the two predators because of species-specific prey demands (Walzer & Schausberger, 2011). The developmental progress of the predators was checked daily. Sex-specific body size differences were used to determine their sex after reaching adulthood. Virgin females and males reared under limited and ample prey supply were termed small and standard-sized females and males, respectively. After the experiment, each female and male was mounted in a drop of Hoyer's medium on a microscope slide (Krantz & Walter, 2009) to measure the dorsal shield length, which is a suitable body size indicator (Croft, Luh, & Schausberger, 1999).

### Mating Behaviour Experiment and Videotaping

First, a small and a standard-sized male of *P. persimilis* or *N. californicus* were placed on the experimental disc. After 20 min a conspecific virgin female, which was either standard-sized or small, was added. To make the three individuals of each triplet discernible for video analyses, they were marked with small water colour dots on their dorsal shields before the experiment. This marking method has been successfully used before and does not affect behaviour (Walzer and Schausberger, 2013a, Walzer and Schausberger, 2013b, Walzer and Schausberger, 2013c, Walzer and Schausberger, 2014). The interactions of the three individuals of each triplet were videotaped for 4 h using a digital camera (Leica DFC495) attached to a stereomicroscope (Leica M5) and the data directly fed into a laptop. The 4 h time span was sufficient to cover the premating behaviours and the complete first mating of both *P. persimilis* and *N. californicus* (Walzer & Schausberger, 2014). Each triplet, defined by species and female body size, was replicated 19–25 times. Each male and female was used only once.

### Video Analyses

Each video was visually analysed to determine mating latency, first mate choice, mating duration and male mating frequency during the experimental period. Males and females were considered to be mating (copulating sensu stricto) when the male was underneath the female in the venter-to-venter position (Amano & Chant, 1978b).

For evaluation of pairwise interactions (proximity, net relative movement) within each triplet, we subjected each complete video (10–15 replicates per species and body size treatment), i.e. videos in which all three individuals remained on the arena throughout the 4 h experimental period, to automated analysis using EthoVision XT 8. The sampling rate during automated analysis was 3.5 samples/s, which was a trade-off between the highest possible sampling rate, the processor speed and the storage capacity of the computer (Bell, 1991). The subtraction method was used for individual detection and discrimination. Proximity was defined as the state in which two individuals were within mutual touching distance and scored when their centres of gravity (the middle of their dorsal shields) were within this predefined distance. The mutual touching distance of 0.5 mm (for *N. californicus*) and 0.7 mm (for *P. persimilis*) was calculated by summing half the dorsal shield length and the length of the first pair of legs of each of the two individuals (Croft et al., 1999, Walzer and Schausberger, 2011). Within this distance the two individuals can easily perceive and recognize each other. For statistical analysis of proximity, the percentage of time spent within this distance was used. Relative movement was defined as the relative displacement between two tracked individuals, whereby the moving speed and direction of both individuals were taken into account to determine the time spent by one individual (the actor) moving to and away from another (the receiver). To exclude random changes in speed and direction of the individuals, unrelated to mating behaviour, these movements were only scored when the distance between the actor and receiver was ≤2 mm. For analysis, net relative movement was calculated by subtracting the time spent by the actor moving away from the receiver from the time spent moving to the receiver. Accordingly, a positive value in net relative movement indicates that the actor approached the receiver, whereas a negative value in net relative movement indicates that the actor moved away from, i.e. avoided, the receiver. Consequently, this unidirectional parameter allowed us to disentangle the relative strengths of male–male competition, female choice and female mating avoidance (Table 1).

**Table 1:** Premating behaviour based on net relative movement direction

Actor	Receiver	Movement direction	Indication
Male	Female	+	Exploitation mate competition
		−	Male mating avoidance
Male	Male	+	Interference mate competition
		−	Avoidance of rival
Female	Male	+	Female choice
		−	Female mating avoidance

The table categorizes possible pairwise net relative movements within triplets of two males competing for a female and gives the associated indication for premating behaviour.

### Statistical Analyses

We used SPSS 18.0.1 (SPSS Inc. Chicago, IL, U.S.A.) for all statistical analyses. Separate generalized linear models (GLM) were used to determine the effects of predator species and prey supply (limited, ample) on female and male body size (normal distribution, identity link function) and the effects of female and male body size (small, standard-sized) on mate choice (binomial distribution, logit link function), mating latency, mating duration and male mating frequency (normal distribution, identity link function). To detail the effects of prey supply on female and male body size within and between predator species, and the effects of female and male body size on mate choice, mating latency, mating duration and male mating frequency within and between the female body size categories, we compared pairs of the estimated marginal means by least significant difference (LSD) tests. For analyses of the interaction parameters, the experimental time was divided into two periods, before and during mating. The interaction parameters of a mating pair were assigned to the female, which can move freely when a male clings to her ventral side (personal observation). Generalized estimation equations (GEEs: normal distribution with identity link function; exchangeable correlation structure among individual pairs) were used to analyse the effects of female body size (small, standard-sized) and individual pair (female and small male, female and standard-sized male, small and standard-sized male) on the proximities and net relative movements for all possible groupings of individuals (groups of three and two before and during mating, respectively, for proximity) and actor–receiver combinations (six and four combinations before and during mating, respectively, for net relative movement). To detail the effects of individual pair or actor–receiver combination on the interaction parameters within and between the female body size categories, we compared pairs of the estimated marginal means by LSD tests. The proportional parameters proximity and net relative movement were arcsine square root transformed before analyses.

## Results

### Male and Female Body Size Plasticity

**Figure 1:**
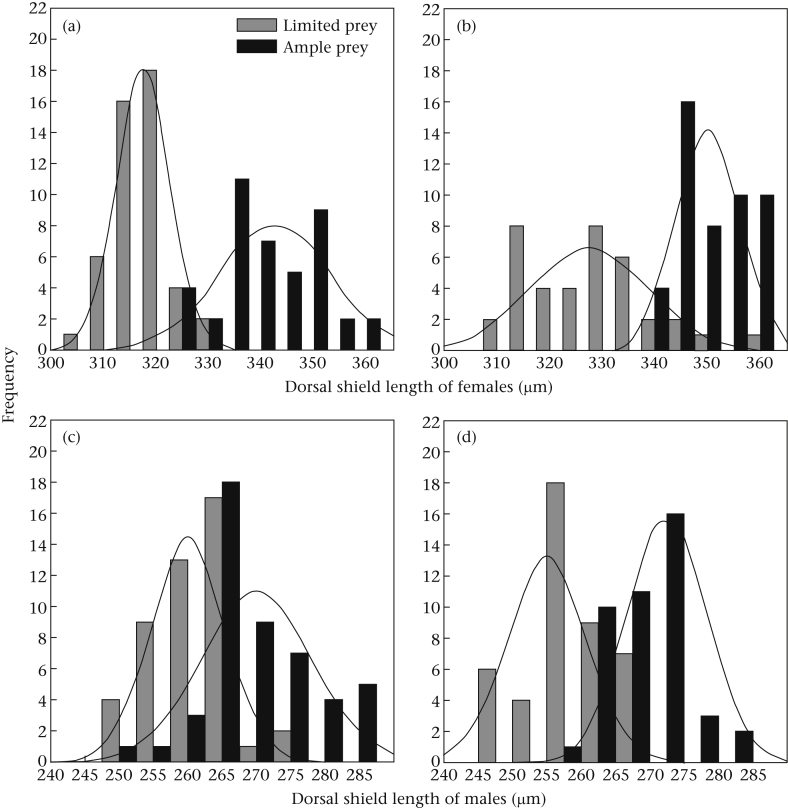
Effects of limited and ample prey during juvenile development on the frequency distribution of body size phenotypes of (a, b) adult females and (c, d) males of (a, c) *P. persimilis* (*N* = 19/22 for females and 41/41 for males reared under limited and ample prey) and (b, d) *N. californicus* (*N* = 20/21 for females and 41/41 for males reared under limited and ample prey).

Differing prey supply during development did not affect survival; all tested females and males reached adulthood. Female body size was affected by prey supply (Wald ${\text{χ}}_{1}^{2}=118.799$, *P* < 0.001) and species (Wald ${\text{χ}}_{1}^{2}=26.088$, *P* < 0.001). Female dorsal shields of *N. californicus* were larger than those of *P. persimilis.* In both species, females reared with limited prey were smaller than females reared with ample prey. The deviations from standard body size were similar in *N. californicus* (6.44%) and *P. persimilis* (7.04%), indicating similar female body size plasticities (prey supply*species interaction: Wald ${\text{χ}}_{1}^{2}=0.066$, *P* = 0.797; Fig. 1a, b). Male body size was affected by prey supply (Wald ${\text{χ}}_{1}^{2}=202.455$, *P* < 0.001) but not species (Wald ${\text{χ}}_{1}^{2}=3.218$, *P* = 0.073). Males grew smaller when reared with limited than with ample prey. Deviation from standard body size was larger in males of *N. californicus* (5.80%) than *P. persimilis* (3.56%; prey supply*species interaction: Wald ${\text{χ}}_{1}^{2}=11.757$, *P* < 0.001), indicating lower male body size plasticity in *P. persimilis* than *N. californicus* (Fig. 1c, d).

### Strength of Male–Male Competition and Female Choice

**Figure 2:**
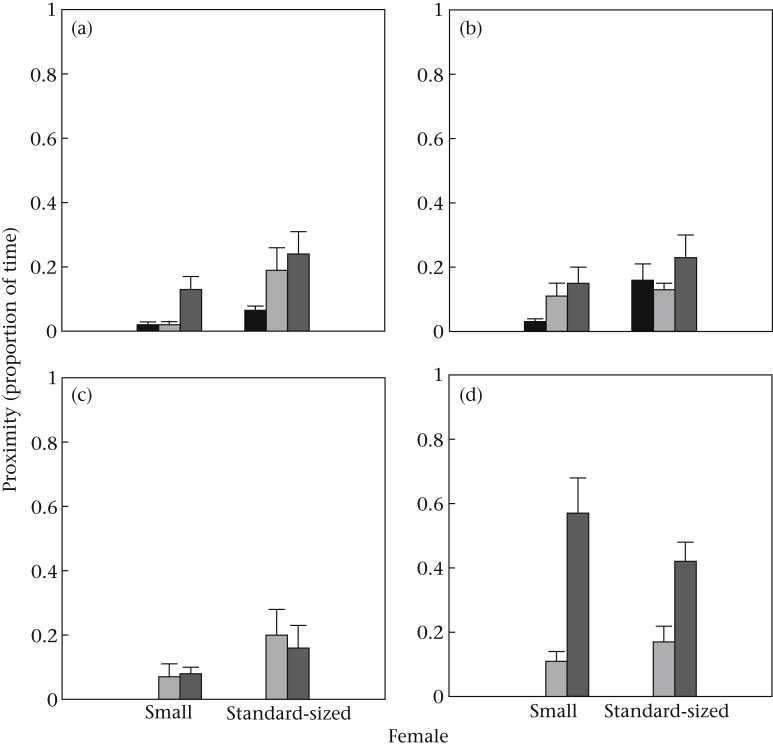
Influence of female body size (small, standard-sized) and individual pair (dark grey bars: female and standard-sized male; light grey bars: female and small male; black bars: small and standard-sized male) on proximity (mean + SE) within triplets of a small and standard-sized male and a small or standard-sized female of (a, c) *P. persimilis* (*N* = 11/10 for standard-sized and small females) or (b, d) *N. californicus* (*N* = 10/12 for standard-sized and small females), (a, b) before mating (interactions among all three individuals) and (c, d) during mating (interaction between mating female and nonmating male).

In *P. persimilis*, female body size (small, standard-sized), individual pair (female and small male, female and standard-sized male, small and standard-sized male) and their interaction affected proximity before mating (Table 2, Fig. 2a). Males and females spent more time in proximity to each other in the presence of the standard-sized than small female (*P* < 0.05 for each individual pair). Small and standard-sized males spent similar times in proximity to the standard-sized female (*P* = 0.449), whereas standard-sized males spent more time in proximity to the small female than did small males (*P* < 0.001; Fig. 2a).

**Table 2:** Influence of female body size and individual pair on proximity within triplets of *P. persimilis* or *N. californicus* in the premating and mating phases

Phase	Factor	*P. persimilis*			*N. californicus*		
		Wald χ²	*df*	*P*	Wald χ²	*df*	*P*
Premating	Female body size	30.139	1	<0.001	15.234	1	<0.001
	Individual pair	27.883	2	<0.001	12.309	2	0.002
	Interaction	6.884	2	0.032	6.384	2	0.041

Mating	Female body size	1.835	1	0.176	0.566	1	0.452
	Individual pair	0.020	1	0.886	47.666	1	<0.001
	Interaction	0.741	1	0.389	3.045	1	0.081

Proximity was analysed before (interactions among all three individuals) and during (interaction between the mating female and the nonmating male) mating using generalized estimation equations (GEE, normal distribution, identity link function). Female body size: small, standard-sized; individual pair: female and standard-sized male, female and small male, small and standard-sized male. Triplets consisted of a standard-sized or small female and a small and standard-sized male.

In *N. californicus*, proximity was affected by female body size, individual pair and their interaction (Table 2, Fig. 2b). Males spent more time in each other's proximity in the presence of the standard-sized than small female (*P* < 0.001). Small and standard-sized males spent similar time in proximity to the females (*P* > 0.160 for both; Fig. 2b).

**Figure 3:**
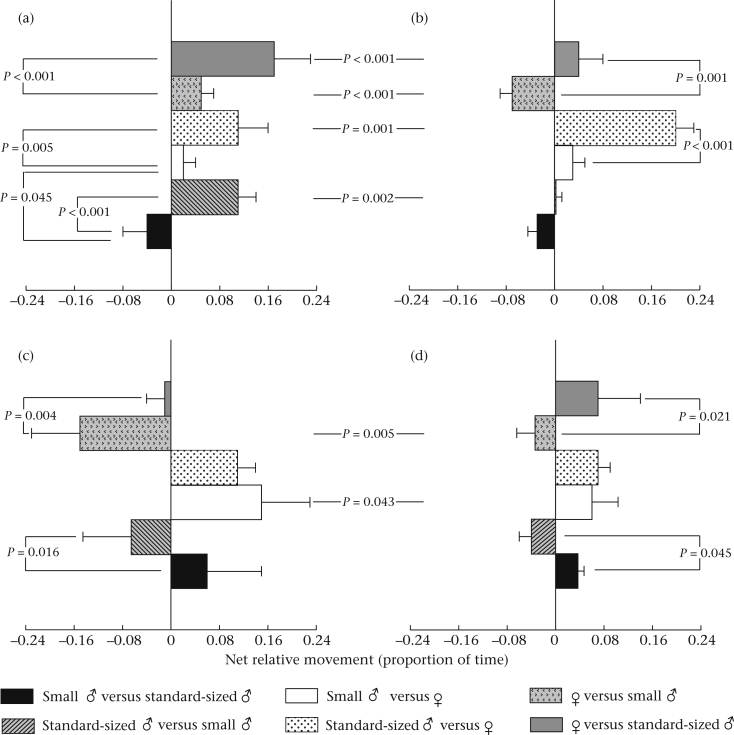
Influence of female body size (small, standard-sized) and actor–receiver combination (female as actor and standard-sized or small male as receiver, small male as actor and standard-sized male or female as receiver, standard-sized male as actor and small male or female as receiver) on the net relative movements (mean + SE) within triplets consisting of a small and standard-sized male and a (a, c) standard-sized or (b, d) small female of (a, b) *P. persimilis* or (c, d) *N. californicus* in the premating period. Negative and positive net relative movement values indicate movements of the actor away from and to the receiver, respectively. *P* values refer to pairwise least significant difference tests following generalized estimating equations; only significant *P* values are given, all other pairwise comparisons were nonsignificant (*P* > 0.05).

In *P. persimilis*, the net relative movements were affected by female body size, actor–receiver combination and their interaction (Table 3). Small males avoided standard-sized males, irrespective of female body size (LSD: *P* = 0.564). Conversely, standard-sized males chased, and occasionally attacked, small males in triplets with standard-sized but not small females (Fig. 3a, b). Small males similarly approached small and standard-sized females (*P* = 0.746). Standard-sized males spent more time approaching the small than standard-sized female (Fig. 3a, b). Standard-sized males were less attractive for small than standard-sized females. Standard-sized females spent less time approaching small than standard-sized males, whereas small females avoided small males (Fig. 3a, b).

**Table 3:** Influence of female body size and actor–receiver combination on net relative movement within triplets consisting of *P. persimilis* or *N. californicus*

Phase	Factor	*P. persimilis*			*N. californicus*		
		Wald χ²	*df*	*P*	Wald χ²	*df*	*P*
Premating	Female body size	9.401	1	0.002	0.353	1	0.553
	Actor-receiver	103.165	5	<0.001	58.621	5	<0.001
	Interaction	38.811	5	<0.001	15.609	5	0.008

Mating	Female body size	0.056	1	0.814	0.001	1	1.000
	Actor-receiver	49.510	3	<0.001	98.035	3	<0.001
	Interaction	5.661	3	0.129	7.867	3	0.049

Triplets consisted of a standard-sized or small female and a small and standard-sized male. Net relative movement was analysed before (interactions among all three individuals) and during (interaction between the mating female and the nonmating male) mating using generalized estimation equations (GEE, normal distribution, identity link function). Female body size: small, standard-sized; actor–receiver combination: actor female and standard-sized male as receiver, actor female and small male as receiver, actor small male and standard-sized male as receiver, actor small male and female as receiver, actor standard-sized male and small male as receiver, actor standard-sized male and female as receiver.

In *N. californicus*, the net relative movements were affected by the actor–receiver combination and its interaction with female body size (Table 3). Small males chased and attacked standard-sized males, which in turn avoided small males (Fig. 3c, d). Irrespective of male and female body sizes, the males always approached the females. The intensity of approaching varied with female body size in small but not standard-sized males. Small males spent more time approaching the standard-sized than small female (Fig. 3c, d). Standard-sized females avoided small males more strongly than did small females. Small females approached standard-sized males, whereas standard-sized females behaved neutrally towards them (Fig. 3c, d).

### Mating Latency, First Mate Choice, Mating Duration and Frequency

**Figure 4:**
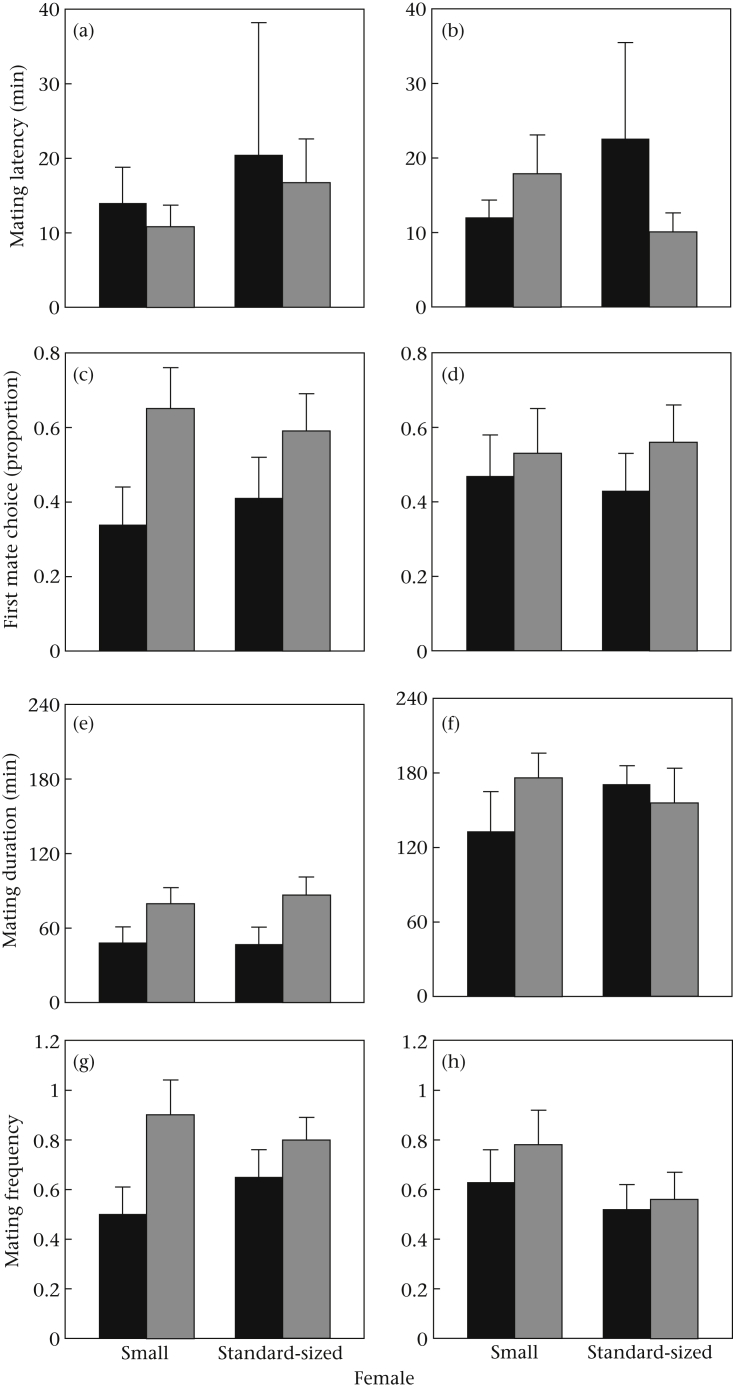
Influence of female and male body size on (a, b) mating latency, (c, d) first mate choice, (e, f) mating duration and (g, h) male mating frequency (mean + SE) within triplets consisting of a small (black bars) and standard-sized (grey bars) male and a small or standard-sized female of (a, c, e, g) *P. persimilis* (*N* = 19/22 for small and standard-sized females ) or (b, d, f, h) *N. californicus* (*N* = 20/21 for small and standard-sized females).

In both species, neither male nor female body size affected the latency to first mating (min; mean ± SE: *P. persimilis*: 15.22 ± 4.08; *N. californicus*: 15.35 ± 3.15; Table 4, Fig. 4a, b).

**Table 4:** Male and female body size effects on mating latency, mate choice, mating duration and mating frequency within triplets of *P. persimilis* or *N. californicus*

Parameter	Factor	*P. persimilis*			*N. californicus*		
		Wald χ²	*df*	*P*	Wald χ²	*df*	*P*
ML (min)	Female body size	0.569	1	0.451	0.053	1	0.819
	Male body size	0.178	1	0.673	0.280	1	0.597
	Interaction	0.001	1	0.972	2.241	1	0.134
MC (%)	Female body size	0.001	1	1.000	0.018	1	0.895
	Male body size	5.229	1	0.022	0.922	1	0.337
	Interaction	0.358	1	0.549	0.049	1	0.825
MD (min)	Female body size	0.050	1	0.823	0.134	1	0.715
	Male body size	7.256	1	0.007	0.348	1	0.555
	Interaction	0.093	1	0.761	1.490	1	0.222
MF	Female body size	0.049	1	0.825	1.953	1	0.162
	Male body size	5.888	1	0.015	0.710	1	0.400
	Interaction	1.217	1	0.270	0.229	1	0.632

ML = mating latency, MC = mate choice, MD = mating duration, MF = mating frequency. The triplets consisted of a small and a standard-sized male competing for a small or standard-sized female. Data were analysed using generalized linear models (GLM, normal distribution and identity link function for ML, MD, MF; bionomial distribution and logit link function for MC).

In *P. persimilis*, first mate choice, mating duration and male mating frequency were only influenced by male body size (Table 4). Of 44 females 27 mated first with the standard-sized male (Fig. 4c), which mated for longer and more often than small males (standard-sized versus small males, mating duration (min, mean ± SE): 83.15 ± 9.62 versus 47.64 ± 8.74; mating frequency: 0.85 ± 0.08 versus 0.58 ± 0.08; Fig. 4e, g).

In *N. californicus*, neither male nor female body size affected the first mate choice (Fig. 4d), mating duration (Fig. 4f) and male mating frequency (Fig. 4h, Table 4).

### Behaviour of Nonmating Male and Mating Female

In *P. persimilis*, neither female body size nor individual pair (mating female and small male, mating female and standard-sized male) affected proximity (Table 2, Fig. 2c).

In *N. californicus*, proximity was only affected by individual pair (Table 2). Pooled over female body sizes, mating females spent more time in proximity (mean proportion ± SE: 0.49 ± 0.06 versus 0.14 ± 0.03) to the nonmating standard-sized than small male (Fig. 2d).

**Figure 5:**
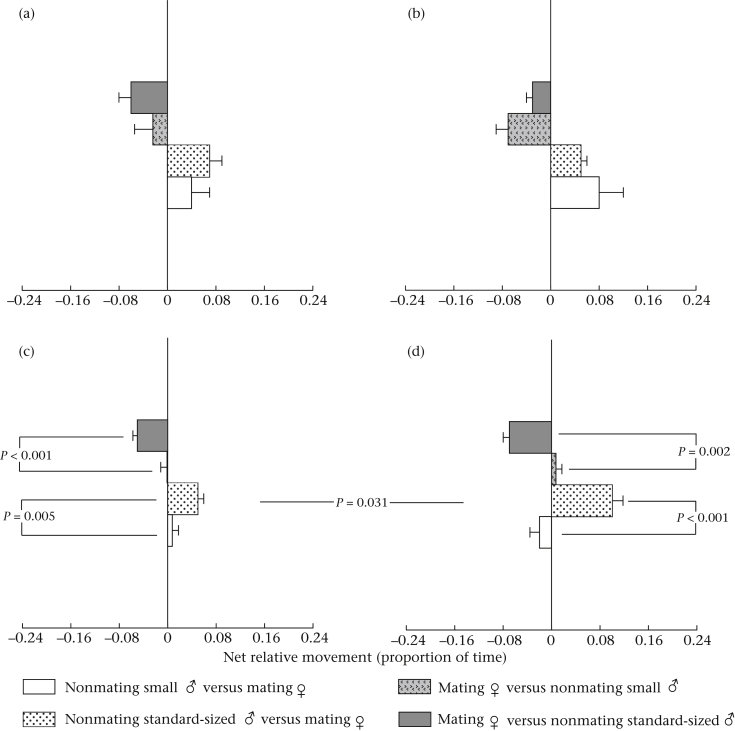
Influence of female body size (a, c = standard-sized; b, d = small) and actor–receiver combination (mating female as actor and standard-sized or small nonmating male as receiver, small nonmating male as actor and mating female as receiver, standard-sized nonmating male as actor and mating female as receiver) on the net relative movements (mean + SE) between a small or standard-sized nonmating male and a mating female of (a, b) *P. persimilis* or (c, d) *N. californicus* during the mating period. Negative and positive net relative movement values indicate movements of the actor away from and to the receiver, respectively. *P* values refer to pairwise least significant difference tests following generalized estimating equations; only significant *P* values are given, all other pairwise comparisons were nonsignificant (*P* > 0.05).

In *P. persimilis*, the net relative movement of the mating female and the nonmating male was only affected by the actor–receiver combination (Table 3, Fig. 5a, b). Pairwise comparisons revealed that the net relative movement patterns were sex-specific (LSD: *P* < 0.001), but unaffected by body size (*P* > 0.800 for both males and females). Mating females avoided nonmating males, whereas nonmating males approached mating females (Fig. 5a, b).

In *N. californicus*, the net relative movement of the mating female and the nonmating male was influenced by the actor–receiver combination, which varied with female body size (Table 3). Small males avoided small mating females, whereas standard-sized males approached small mating females. Standard-sized males approached standard-sized mating females more vigorously than did small males. Additionally, standard-sized nonmating males spent more time approaching the small than the standard-sized mating female. Both small and standard-sized mating females avoided the standard-sized but not small nonmating male (Fig. 5c, d).

## Discussion

Consistent with the adaptive canalization hypothesis (Schmalhausen, 1949, Stearns and Kawecki, 1994, Stillwell et al., 2010, Waddington, 1942), our study reveals that deviations from standard body size result in mating disadvantages in *P. persimilis* but not *N. californicus* males. Male body size is more strongly canalized in *P. persimilis* than *N. californicus* and only in *P. persimilis* did small males experience lower mating success and shorter mating durations than standard-sized males. In *P. persimilis*, both male–male competition and female choice favoured standard-sized over small males. Standard-sized males were superior competitors and preferred by the females as mates. In contrast, male–male competition and female choice conflicted in *N. californicus*. Females preferred standard-sized over small males but small males compensated for this disadvantage by hyperaggressive behaviour towards standard-sized males, leading to similar mating success of small and standard-sized males.

### Species-specific Premating and Mating Behaviour

Female body size decisively influenced the premating interactions in *P. persimilis*. Standard-sized females were more strongly attracted by standard-sized than small males, indicated by differing investments in mate searching behaviour. Also, small females were attracted by standard-sized males but less so than standard-sized females. Additionally, small females strongly avoided small males. Ultimately, avoiding small male mates in choice situations is adaptive because they are detrimental to female fecundity (Walzer & Schausberger, 2014). Standard-sized females did not avoid small males, which is probably because of size-dependent abilities to resist male mating attempts upon encounter. Avoiding small males by small females was traded off against time spent approaching standard-sized males, resulting in shorter stays in proximity to standard-sized males, as compared to standard-sized females. Body size-specific female behaviour, in turn, shifted the tactics of standard-sized but not small males to get access to receptive females. Standard-sized males displayed strong interference mate competition only in the presence of standard-sized but not small females. Although small males behaved qualitatively and quantitatively similarly in the presence of small and standard-sized females (rival avoidance, approaching the female), they spent more time in proximity to the standard-sized females because of the opposing female responses towards them (avoidance by small females, approaching by standard-sized females). An appropriate response of the standard-sized males in this scenario was chasing and trying to displace the small male from the standard-sized female's proximity, i.e. to succeed in interference competition. The small female, however, invested more time in running away from the small male than approaching the standard-sized male. In such a scenario, the challenge for the standard-sized male was outracing the rival in locating the female, i.e. to succeed in exploitation (scramble mate) competition. Consequently, standard-sized males spent more time in proximity to small females than did small males.

Mating itself was also negatively affected by small male body size because small *P. persimilis* males mated for less time than standard-sized males. Mating duration correlates with the quantity of sperm transferred in several species (Arnqvist and Danielsson, 1999, Wilder and Rypstra, 2007) including *P. persimilis* (Amano & Chant, 1978b). Thus, it could be that females mating with small males finished the mating earlier to avoid potential fitness costs such as lower numbers of fertilized eggs, or perhaps small males had less sperm material available than standard-sized males and therefore finished mating earlier. However, the mating duration of small males was twice as long (Walzer & Schausberger, 2014) in the absence than in the presence of a competitor, making these two explanations unlikely. Most likely, the short mating duration of small males was caused by disturbance of the larger competitor. Standard-sized males were more successful in mating disruption than small males.

Standard-sized *N. californicus* males were attracted by both small and standard-sized females. Females preferred standard-sized over small males as mates: small females approached standard-sized but avoided small males and standard-sized females strongly avoided small but not standard-sized males. If male–male competition and female choice were aligned, standard-sized males would have been the first mates. However, the likelihood of being the first mate did not vary with male body size, which was obviously due to the more aggressive behaviour of the small males towards the larger rivals than vice versa. Small males chased and attacked standard-sized males particularly in the presence of standard-sized females. Hyperaggressive behaviour of small against large male competitors has been also observed in fishes, reptiles and crustaceans (Jenssen et al., 2005, Moretz, 2003, Smith et al., 1994, Svensson et al., 2012) and is termed the ‘Napoleon complex’ (Just & Morris, 2003). Explanations for this behaviour include (1) the desperado effect, that is, small males initiate fights because they have no other opportunities to get access to receptive females, (2) the perception error effect, that is, small males launch attacks because they overestimate their fighting abilities, and (3) the precedence effect, that is, small males attack first because the chance of winning the fight is higher for the initiator than the reactor (Just and Morris, 2003, Morrell et al., 2005). Whichever explanation applies to *N. californicus*, owing to their hyperaggressive behaviour small males were the superior interference mate competitors, reducing the likelihood of encounter between the females and the standard-sized males and thereby counterbalancing the female preference for standard-sized males. This conclusion is strongly supported by the proximity data. Without interference by small males, the standard-sized males would have spent more time in proximity to the females than their smaller rivals because of the female preference for standard-sized males. Owing to interference by small males, small and standard-sized males spent similar times near the females. Thus, the opposing directions of male–male competition and female choice in *N. californicus* indicate sexual conflict between small males and females (Chapman, Arnqvist, Bangham, & Rowe, 2003). Male body size does not affect the LRS of female *N. californicus* (Walzer & Schausberger, 2014) but could affect other traits important to fitness such as offspring quality.

In both species, interference male competition for mates was more intense in the presence of standard-sized than small females, indicated by the time spent in each other's proximity. Positive correlations between female body size and intrasexual male aggressiveness are also known from other arthropods such as dung flies (Sigurjonsdottir & Parker, 1981) and autumn spiders (Hack, Thompson, & Fenandes, 1997). Proximately, female body size may correlate with the quantity and quality of their sex pheromones, making larger females more attractive and consequently intensifying male–male competition. Ultimately, larger male investment in competition for standard-sized females pays because of the fecundity advantage of standard-sized over small females (Andersson, 1994, Blanckenhorn, 2005, Walzer and Schausberger, 2013c).

### Conclusion

Our study provides a key example of the implications of sexually selected body size plasticity on mating behaviour and disentangles the relative strengths of male–male competition and female choice.

Mate preference for standard-sized males by *P. persimilis* but not *N. californicus* females seems closely linked to the species-specific levels of polyandry (low level in *P. persimilis*, medium level in *N. californicus*). *Neoseiulus californicus* females are likely to be less choosy than *P. persimilis* females because mate acceptance errors such as first choosing a poor-quality male (possibly indicated by small body size) can be more easily compensated for by remating.

Disentangling the effects of body size and its plasticity on simultaneously operating male–male competition and female choice, and their relative contribution to mating success in competitive situations, respectively, is a methodological challenge and almost impossible with direct visual observation. Hence, the majority of empirical studies have focused on one of the two forces, although such an approach provides an incomplete view of sexual selection acting on body size (Hunt, Breuker, Sadowski & Moore, 2009). This is especially true under sexual conflict with opposing interests of superior male competitors and mate-choosing females (Wong & Candolin, 2005). Our set-up of videotaping competitive mating situations and subsequent automated video analysis of individual behaviours shows how to meet this methodological challenge and successfully disentangle the body size effects on male–male competition and female choice.
